# Probiotics in critically ill children

**DOI:** 10.12688/f1000research.7630.1

**Published:** 2016-03-29

**Authors:** Sunit C. Singhi, Suresh Kumar

**Affiliations:** 1 Deptartment of Pediatrics, MM Institute of Medical Science and Research, Mullana, 133207, India; 2 Department Of Pediatrics, Advanced Pediatrics Centre, Post graduate Institute of Medical Education and Research, Chandigarh, 160012, India

**Keywords:** Antibiotic associated Diarrhea, Candida colonization, candidemia, Critical illness, Critically ill children, Nosocomial Infections, Probiotics, Ventilator Associated Pneumonia

## Abstract

Gut microflora contribute greatly to immune and nutritive functions and act as a physical barrier against pathogenic organisms across the gut mucosa. Critical illness disrupts the balance between host and gut microflora, facilitating colonization, overgrowth, and translocation of pathogens and microbial products across intestinal mucosal barrier and causing systemic inflammatory response syndrome and sepsis. Commonly used probiotics, which have been developed from organisms that form gut microbiota, singly or in combination, can restore gut microflora and offer the benefits similar to those offered by normal gut flora, namely immune enhancement, improved barrier function of the gastrointestinal tract (GIT), and prevention of bacterial translocation. Enteral supplementation of probiotic strains containing either *Lactobacillus* alone or in combination with *Bifidobacterium* reduced the incidence and severity of necrotizing enterocolitis and all-cause mortality in preterm infants. Orally administered *Lactobacillus casei* subspecies *rhamnosus*, *Lactobacillus reuteri*, and *Lactobacillus rhamnosus* were effective in the prevention of late-onset sepsis and GIT colonization by *Candida* in preterm very low birth weight infants. In critically ill children, probiotics are effective in the prevention and treatment of antibiotic-associated diarrhea. Oral administration of a mix of probiotics for 1 week to children on broad-spectrum antibiotics in a pediatric intensive care unit decreased GIT colonization by *Candida*, led to a 50% reduction in candiduria, and showed a trend toward decreased incidence of candidemia. However, routine use of probiotics cannot be supported on the basis of current scientific evidence. Safety of probiotics is also a concern; rarely, probiotics may cause bacteremia, fungemia, and sepsis in immunocompromised critically ill children. More studies are needed to answer questions on the effectiveness of a mix versus single-strain probiotics, optimum dosage regimens and duration of treatment, cost effectiveness, and risk-benefit potential for the prevention and treatment of various critical illnesses.

## Introduction

Critically ill patients are predisposed to altered gut microflora, which can lead to infective and non-infective complications and adverse outcome ^1– 3^. Probiotic bacteria have the potential to restore the balance of gut microflora in critically ill children and confer a health benefit when given for various indications. Probiotics are defined by a joint working group of the Food and Agriculture Organization of the United Nations/World Health Organization as “live microbes which when administered in adequate amount confer health benefit to the host” ^4^. In addition, probiotics should be non-pathogenic, stable in acid and bile, able to adhere to and colonize human gut mucosa, and retain viability during storage and use. They should be scientifically demonstrated to have beneficial physiological effects and safety so that they can be used to improve microbial balance and to confer health benefit. In recent years, probiotics have been increasingly used in critical care settings for the prevention of certain diseases that are otherwise associated with high mortality. In this review, we examine the current status of probiotics in the care of critically ill children on the basis of available literature and identify directions for future research.

## Gut microflora

The human gut represents a complex ecosystem where a delicate balance exists between the host and the microflora. More than 400 different species of microbes live in the gut as commensal; the total estimated number is more than 10 times the number of eukaryotic cells in the human body ^3, 5^. Human gut microflora consists principally of obligate anaerobes (95%; *Bifidobacterium*, *Clostridium*, *Eubacterium*, *Fusobacterium*, *Peptostreptococcus*, and *Bacteriodes*) and facultative anaerobes (1–10%; *Lactobacillus*, *Escherichia coli*, *Klebsiella*, *Streptococcus*, *Staphylococcus*, and *Bacillus*). *Bifidobacteria* are predominant microbes that represent up to 80% of the cultivable fecal bacteria in infants and 25% in adults. Each human being has his or her own unique microbial composition, especially of lactic acid bacterial (LAB) strains ^3^. Most of these microbes have health-promoting effects; however, a few are potentially pathogenic. Normally, the ‘good’ microbes outnumber potentially pathogenic bacteria and live in symbiosis with the host. The optimal balance, composition, and function of gut microflora depend on the supply of food (fermentable fibers and complex proteins) and fluctuate with antibiotic usage, diarrheal diseases, and critical illness ^3^. The gut microflora benefits the host by performing various crucial functions ( Table 1).

**Table 1.** Beneficial functions performed by gut microbiota.

Beneficial functions	Details of beneficial functions
Immune response	Gut microflora stimulate the proliferation and differentiation of epithelial cells in large and small intestines, modulate innate and adaptive immune response and development of competent gut- associated immune system, and maintain an immunologically balanced inflammatory response ^5, 61, 62^.
Physical barrier function (colonization resistance)	Gut microbiota provide a physical barrier against pathogen invasion by competing for epithelial cell adhesion sites, preventing epithelial invasion, competing for available nutrients affecting the survival of potential pathogens, and producing anti-bacterial substances (e.g. bacteriocins and lactic acid), making the environment unsuitable for the growth of pathogens ^3, 63^.
Nutritive functions	Gut microbiota produce several enzymes for fermentation of non-digestible dietary residue and endogenously secreted mucus and help in recovering lost energy in the form of short-chain fatty acids ^64^. They also help in the absorption of calcium, magnesium, and iron; synthesis of vitamins (folic acid and vitamin B1, B2, B3, B12, and K); biotransformation of bile acids; and conversion of pro-drugs to active metabolites ^64– 66^.

## Critical illness and gut microflora

Critical illness and its treatment create a hostile environment in the gastrointestinal tract (GIT) and alter the microflora that tilts the balance to favor overgrowth of pathogens. The hostile environment is exacerbated by the use of broad-spectrum antibiotics, invasive central lines, endotracheal intubation, mechanical ventilation, antacids, H _2_ blockers, steroids, and immunosuppressive and cytotoxic therapy. Multiple organ dysfunction syndrome (MODS), burns, malnutrition, changes in nutrient availability, gut motility, pH, redox state, osmolality, and the release of high amounts of stress hormones (including catecholamines) further compromise the critical balance ^2, 3^.

Studies in experimental models have shown that after onset of acute pancreatitis there was disappearance of beneficial LAB within 6 to 12 hours ^6– 8^. In patients with systemic inflammatory response syndrome (SIRS), there is a reduction in beneficial bacteria ( *Bifidobacterium* and *Lactobacillus*) that leads to a decrease in short-chain fatty acid levels and elevation of intestinal pH, indicating a disturbed intestinal environment ^9^. Hostile gut environment and disruption of the balance of gut microflora alter local defense mechanisms and lead to colonization and overgrowth of potentially pathogenic commensals such as *Salmonella*, *E. coli*, *Yersinia*, and *Pseudomonas aeruginosa*. These pathogenic commensals cause cytokine release, cell apoptosis, activation of neutrophils, and disruption in epithelial tight junctions ^1, 2^. With loss of “colonization resistance”, the gut is unable to prevent the translocation of pathogens and toxins across the gut wall into the bloodstream, leading to SIRS, MODS, and mortality. Interestingly, the gut has been identified as the originator and promoter of health care-associated infections (HCAIs) and MODS in critically ill patients ^1, 10^. Restoring the beneficial gut microflora with an exogenous supply of new and effective microbes (probiotics) seems an attractive option to restore the “colonization resistance”.

## Commonly used probiotics

The most frequently used probiotic strains are *Lactobacillus* and *Bifidobacterium* ^11^; other species of probiotics are enlisted in Table 2. These probiotics are used either singly or in combination. Multi-strain probiotics are likely to be better than single-strain probiotics, as individual probiotics have different functions and have synergistic effects when administered together. A daily intake of 10 ^6^–10 ^9^ colony-forming units (CFUs) is reportedly the minimum effective dose for therapeutic purposes ^11, 12^.

**Table 2.** Microbial species commonly used for designing probiotic strains.

Species	Examples
*Lactobacillus* species	*L. acidophilus*, *L. casei*, *L. rhamnosus*, *L. reuteri*, *L. paracasei*, *L. lactis*, *L. plantarum*, and *L. sporogenes*
*Bifidobacterium* species	*B. bifidum*, *B. bifidus*, *B. longum*, and *B. lactis*
*Enterococcus* species	*E. faecalis* and *E. faecium*
*Saccharomyces*	*S. boulardii* and *S. cerevisiae*
Others	*Streptococcus thermophilus*, *Escherichia coli*, *Bacillus* spp., and *Enterococcus* spp.

## Mechanism of beneficial effects of probiotics

The beneficial effects of probiotics are due to change in the composition of gut flora and modification of immune response ^13^. Probiotic strains activate mucosal immunity and stimulate cytokine production, IgA secretion, phagocytosis, and production of substances (such as organic acids, hydrogen peroxide, and bacteriocins) that are inhibitory to pathogens. They also compete for nutrients with pathogenic bacteria and inhibit pathogen attachment and action of microbial toxin. Probiotics also have a trophic effect on intestinal mucosa (by stimulating the proliferation of normal epithelium that maintains mucosal barrier defenses), modulate innate and adaptive immune defense mechanisms via the normalization of altered gut flora, and prevent bacterial translocation ^12– 16^. Table 3 and Table 4 provide a summary of various studies demonstrating different mechanisms of action of probiotics in experimental and clinical studies, respectively.

**Table 3.** Experimental studies showing mechanisms of beneficial effects of probiotics.

Mechanism of action	Authors	Experimental group	Intervention	Outcome
Probiotics maintain healthy flora and reduce the growth of pathogens and colonization.	Jiang *et al.* ^39^	Opportunistic oral *Candida albicans*	*Lactobacillus rhamnosus* GG, *Lactobacillus casei Shirota*, *Lactobacillus reuteri* SD2112, *Lactobacillus brevis* CD2, *Lactobacillus bulgaricus* LB86, and *Lactobacillus bulgaricus* LB Lact	*L. rhamnosus* GG had inhibitory activity against *Candida glabrata*. None had inhibitory activity against *Candida krusei*.
	Machairas *et al.* ^67^	Experimental infection of mice by multi-drug resistant *Pseudomonas* *aeruginosa* and *Escherichia coli*	Pretreatment with *Lactobacillus* *plantarum* and commercial preparation of four probiotics: *L. plantarum*, *Lactobacillus* *acidophilus*, *Saccharomyces* *boulardii*, and *Bifidobacterium* *lactis* LactoLevure *®*; Uni-Pharma S.A. Attica, Greece	*L. plantarum* pretreatment significantly increased survival after challenge by either *P. aeruginosa* (66.7% versus 31.3%; *P* = 0.026) or *E. coli* (56% versus 12%, *P* = 0.003). Survival benefit was even more pronounced with LactoLevure ^®^.
Probiotics prevent bacterial translocation.	Mangell *et al.* ^68^	Endotoxemia rat model	Pretreatment with *L. plantarum* 299v for 8 days	*L. plantarum* 299v pretreatment reduced bacterial translocation to 0% and 12% in mesenteric lymph nodes and liver, respectively.
	Ruan *et al.* ^69^	In hemorrhagic-shock rat model	Pretreated with phosphate-buffered saline (PBS), *Bifidobacteria*, or microencapsulated *Bifidobacteria*	Pretreatment with encapsulated *Bifidobacteria* reduced incidence of bacterial translocation to mesenteric lymph nodes compared with PBS (40% versus 80%, *P* <0.05). Non-significant reduction in bacterial translocation by intact *Bifidobacteria* when compared with PBS control (55% versus 80%, *P* >0.05).
	Sánchez *et al.* ^70^	In rats with carbon tetrachloride-induced cirrhosis	VSL#3	Decreased incidence of bacterial translocation in VSL#3 group than in water group (8% versus 50%; *P* = 0.03)

**Table 4.** Clinical studies showing mechanisms of beneficial effects of probiotics.

Mechanism of action	Authors	Patient group	Intervention	Outcome
Probiotics maintain healthy flora and reduce the growth of pathogens and colonization.	Shimizu *et al.* ^71^	Randomized controlled trial (RCT) involving patients with systemic inflammatory response syndrome (SIRS) (n = 29)	*Bifidobacterium breve*, *Lactobacillus casei*, and galacto-oligosaccharide	Probiotic group had significantly greater levels of beneficial *Bifidobacterium*, *Lactobacillus*, and organic acids in the gut. The incidences of infectious complications were significantly lower in the probiotic group (enteritis 7% versus 46%; pneumonia 20% versus 52%; bacteremia 10% versus 33%).
	Hayakawa *et al.* ^72^	RCT involving mechanically ventilated patients (n = 47)	Synbiotic ( *Lactobacillus*, *Bifidobacterium*, and galacto- oligosaccharides) for 8 weeks	Synbiotic group had significantly increased *Bifidobacterium* and *Lactobacillus* (to 100 times the initial level), increased acetic acid concentration (71.1±15.9 versus 46.8±24.1μmol/g), decreased pH, decreased Gram-negative rod (to one-tenth of the initial level) in the gut, and decreased *Pseudomonas* *aeruginosa* in the lower respiratory tract when compared with the control group.
	Jain *et al.* ^73^	RCT involving intensive care unit (ICU) patients (n = 90)	Multi-strain synbiotic for 7 days	Synbiotic group had lower incidence of potentially pathogenic bacteria (43% versus 75%, *P* = 0.05) and multiple organisms (39% versus 75%, *P* = 0.01) in nasogastric aspirates than controls.
	Mohan *et al.* ^74^	RCT including preterm neonates (n = 69)	*Bifidobacterium lactis* Bb12 for 7–21 days	Probiotic group had higher counts of *Bifidobacterium* (log10 values per grams of fecal wet weight: 8.18±0.54 versus 4.82±0.51; *P* = 0.001); and lower counts of *Enterobacteriaceae* (7.80±0.34 versus 9.03±0.35; *P* = 0.015) and *Clostridium* spp. (4.89±0.30 versus 5.99±0.32; *P* = 0.014) than in placebo group.
	Manzoni *et al.* ^32^	RCT including very low birth weight preterm babies (n = 80)	*L. casei rhamnosus* for 6 weeks	Reduced incidence of *Candida* colonization in gut in probiotic group as compared with placebo group (23.1% versus 48.8%; *P* = 0.01).
Probiotics reduce inflammation	Sanaie *et al.* ^75^	RCT involving critically ill patients (n = 40)	VSL#3 *(Lactobacillus*, *Bifidobacterium*, and *Streptococcus* *thermophilus*) for 7 days	Reduced inflammation (reduced acute physiology and chronic health evaluation II [APACHE II] score, sequential organ failure assessment [SOFA], interleukin-6 [IL-6], procalcitonin, and protein)
	McNaught *et al.* ^76^	RCT involving critically ill patients (n = 103)	*L. plantarum 299v*	Late attenuating effect (after 15 days) on SIRS (as measured by serum IL-6 levels)
	Ebrahimi- Mameghani *et al.* ^77^	RCT involving ICU cases (n = 40)	VSL#3 use for 7 days	Reduction in inflammation (C-reactive protein and APACHE II score). No significant change in markers of oxidative stress:total antioxidant capacity (TAC) and malondialdehyde (MDA) levels.

## Probiotic use in critically ill children

Studies have evaluated the role of probiotics in critically ill children for the prevention and treatment of necrotizing enterocolitis (NEC), antibiotic-associated diarrhea (AAD), and HCAIs, including ventilator-associated pneumonia (VAP), *Candida* colonization, and invasive candidiasis.

### Probiotics and necrotizing enterocolitis

In 1999, a study showed that oral administration of *Lactobacillus acidophilus* and *Bifidobacterium infantis* reduced NEC ^17^. This was followed by a negative study showing that 7 days of *L. rhamnosus* GG supplementation starting with the first feed was not effective in reducing the incidence of urinary tract infection, NEC, or sepsis in preterm infants ^18^. However, subsequent randomized controlled trials (RCTs) with different strains of *Lactobacilli* and *Bifidobacteria* showed a significant reduction in the development of NEC ^19, 20^. A systematic review and meta-analysis by Alfaleh *et al.* ^21^ in 2008 concluded that probiotic supplementation reduced the incidence of NEC stage II (or more) and mortality. A more recent meta-analysis by the same authors, involving 24 trials in preterm neonates, found that supplementation with probiotic preparations containing *Lactobacillus* either alone or in combination with *Bifidobacterium* prevents severe NEC and reduces all-cause mortality ^22^.

### Probiotics in antibiotic-associated diarrhea

The osmotic and invasive AAD is often observed among critically ill children receiving broad-spectrum antibiotics. It is attributed to overgrowth of pathogens and a decrease in population of microbes that have beneficial metabolic functions ^23^. Several investigators have shown that probiotics could prevent AAD. The results of meta-analyses on the effect of probiotics for the prevention of AAD are given in Table 5.

**Table 5.** Findings of various meta-analyses of studies addressing the effect of probiotics on antibiotic-associated diarrhea.

Authors (year)	Number of trials	Number of subjects	Results
D’Souza *et al.* ^78^ (2002)	Nine randomized controlled trials (RCTs), including two pediatric RCTs	1214	Probiotics were effective in the prevention of antibiotic- associated diarrhea (AAD) (odds ratio [OR] 0.37, 95% confidence interval [CI] 0.26–0.53, *P*<0.001). *Saccharomyces boulardii* and *Lactobacilli* had the best potential.
Szajewska *et al.* ^79^ (2006)	Six pediatric RCTs	766	Treatment with probiotics compared with placebo reduced the risk of AAD from 28.5% to 11.9% (risk ratio [RR] 0.44, 95% CI 0.25–0.77).
Johnston *et al.* ^80^ (2006)	Six pediatric RCTs	707	Probiotics resulted in significant reduction in the incidence of AAD (RR 0.43, 95% CI 0.25–0.75).
Hempel *et al.* ^81^ (2012)	63 RCTs, all ages	11,811	Probiotics associated with significant reduction in AAD (RR 0.58, 95% CI 0.50–0.68, *P*<0.001).
Szajewska *et al.* ^82^ (2015)	21 RCTs involving children and adults	4780	*S. boulardii* compared with placebo or no treatment reduced risk of AAD from 18.7% to 8.5% (RR 0.47, 95% CI 0.38–0.57). In children, from 20.9% to 8.8% (six RCTs, n = 1653, RR 0.43, 95% CI 0.3–0.6). In adults, from 17.4% to 8.2% (15 RCTs, n = 3114, RR 0.49, 95% CI 0.38–0.63).
Szajewska *et al.* ^83^ (2015)	12 RCTs involving children and adults	1499	*Lactobacillus rhamnosus* GG compared with placebo or no additional treatment reduced risk of AAD from 22.4% to 12.3% (RR 0.49, 95% CI 0.29–0.83).

### Probiotics for the prevention of health care-associated infections

There are limited studies in this field in critically ill children. Most of the studies are in critically ill adults. These studied have yielded mixed results. A randomized trial that included mechanically ventilated, multiple-trauma patients (n = 65) demonstrated that 15 days of multi-strain probiotic therapy led to a significant reduction in the rate of infection, SIRS, severe sepsis, duration of ventilation, intensive care unit (ICU) stay, and mortality ^24^. In contrast, a systematic review (eight RCTs; n = 999) revealed no beneficial effect of probiotics or synbiotics on critically ill adults in terms of clinical outcomes, namely length of ICU stay, incidence of HCAIs, pneumonia, and hospital mortality ^25^. A meta-analysis of 12 RCTs that included 1546 critically ill adult patients found that the use of probiotics was associated with a statistically significant reduction in nosocomial pneumonia (odds ratio [OR] = 0.75, 95% confidence interval [CI] = 0.57–0.97, *P* = 0.03, I[2] = 46%), although there was no statistically significant effect on ICU and in-hospital mortality and duration of ICU and hospital stay ^26^. In the same year, another systemic review of 23 RCTs, by Petrof *et al.* ^27^, involving critically ill adults, demonstrated that probiotics were associated with reduced infectious complications (risk ratio = 0.82, 95% CI = 0.69–0.99; *P* = 0.03; test for heterogeneity *P* = 0.05; I = 44%), VAP rates (risk ratio = 0.75, 95% CI = 0.59–0.97; *P* = 0.03; test for heterogeneity *P* = 0.16; I = 35%), and ICU mortality (risk ratio = 0.80, 95% CI = 0.59–1.09; *P* = 0.16; test for heterogeneity *P* = 0.89; I = 0%). There was no influence on in-hospital mortality or length of ICU and hospital stay. The results of a meta-analysis by Bo *et al.* ^28^ that included eight RCTs (n = 1083) in adults found that probiotics resulted in decreased incidence of VAP (OR = 0.70, 95% CI = 0.52–0.95, low-quality evidence).

In critically ill children, Honeycutt *et al.* ^29^ observed a statistically non-significant trend toward an increased rate of infection with probiotic strain (11 versus 4, relative risk [RR] = 1.94, 95% CI 0.53–7.04; *P* = 0.31). They had randomly assigned 61 critically ill children to receive either a probiotic (one capsule of *L. rhamnosus* strain GG and inulin daily) or placebo (one capsule of inulin) until discharge from the hospital. However, these findings were not substantiated by subsequent studies in children. Wang *et al.* ^30^, in an RCT comprising 100 critically ill full-term infants, found that administration of a probiotics mix ( *L. casei*, *L. acidophilus*, *Bacillus subtilis*, and *Enterococcus faecalis*) three times daily for 8 days enhanced immune activity, decreased incidence of nosocomial pneumonia and MODS, and reduced length of hospital stay. Recently, Banupriya *et al.* ^31^ published an open-label randomized trial that included 150 children, aged 12 years or younger, who were likely to need mechanical ventilation for more than 48 hours. The intervention group received a probiotics mix of *L. acidophilus*, *L. rhamnosus*, *Lactobacillus plantarum*, *L. casei*, *Lactobacillus bulgaricus*, *Bifidobacterium longum*, *B. infantis*, *Bifidobacterium breve*, and *Streptococcus thermophilus* for 7 days or until discharge, whichever was earlier; the controls did not receive either probiotics or any placebo. The authors found that probiotics resulted in a significant decrease in incidence of VAP, duration of pediatric ICU (PICU) and hospital stay, and mechanical ventilation. Also, the probiotic group had lower colonization rates with potentially pathogenic organisms ( *Klebsiella* and *Pseudomonas*) (34.3% versus 51.4%; *P* = 0.058) and reductions of VAP caused by *Klebsiella* (4.2% versus 19.4%, *P* = 0.01) and *Pseudomonas* (4.2% versus 16.7%, *P* = 0.03). There were no complications due to the administration of probiotics.

### Probiotic use, candida colonization, and invasive candidiasis

Several RCTs have addressed the role of probiotics in the prevention of *Candida* colonization and invasive candidiasis in neonates. Manzoni *et al.* ^32^, in an RCT involving 80 very low birth weight (VLBW) neonates, demonstrated that orally administered *L. casei* subspecies *rhamnosus* significantly reduced the incidence and the intensity of enteric colonization by *Candida* species. Romeo *et al.* ^33^, in a study of 249 preterm neonates who were subdivided to receive *L. reuteri* (n = 83), *L. rhamnosus* (n = 83), and no supplementation (n = 83), found that both the probiotics were effective in reducing *Candida* colonization in the GIT, late-onset sepsis, and abnormal neurological outcomes. Another RCT, by Demirel *et al.* ^34^, found that in VLBW infants (gestational age of not more than 32 weeks and birth weight of not more than 1500g) prophylactic *Saccharomyces boulardii* supplementation was as effective as nystatin in reducing fungal colonization and invasive fungal infection and was more effective in reducing the incidence of clinical sepsis and number of sepsis attacks. An RCT by Roy *et al.* ^35^ demonstrated that supplementation with a mix of multiple probiotics (a mix of *L. acidophilus*, *B. longum*, *Bifidobacterium bifidum*, and *Bifidobacterium lactis*) in preterm infants and neonates led to reduced enteral fungal colonization and invasive fungal sepsis, earlier establishment of full enteral feeds, and reduced duration of hospital stay. More recently, Oncel *et al.* ^36^, in a RCT, demonstrated that prophylactic oral administration of *L. reuteri* in preterm infants (gestational age of not more than 32 weeks and birth weight of not more than 1500g) was as effective as nystatin in the prevention of fungal colonization and invasive candidiasis and reduced the incidence of sepsis, feeding intolerance, and duration of hospitalization.

Limited data are available on the role of probiotics in the prevention of *Candida* colonization and *Candida* infection in critically ill pediatric patients. In a placebo-controlled RCT, we found that administration of a mix of probiotics ( *L. acidophilus*, *L. rhamnosus*, *B. longum*, *B. bifidum*, *S. boulardii*, and *S. thermophilus*) for 1 week to children being treated in a PICU with broad-spectrum antibiotics decreased the prevalence of *Candida* colonization of the GIT by 34.5% and 37.2% on days 7 and 14, respectively, and led to an almost 50% reduction in the incidence of candiduria ^37^. We also observed that the rate of *Candida* bloodstream infection was lower in the probiotic group as compared with the placebo group; the difference, however, was not statistically significant, as the sample size was not sufficient to evaluate this outcome. To test the hypothesis that the enteral supplementation with probiotics in critically ill children can decrease the prevalence of invasive candidiasis, we conducted a retrospective “before and after” study that included critically ill children on broad-spectrum antibiotics for at least 48 hours. The study showed that the probiotics group (4 of 344, 1.2%) had a significantly lower incidence of candidemia than the control group (14 of 376, 3.7%, RR 0.31; 95% CI 0.10–0.94; *P* = 0.03) ^38^. Candiduria was noted in 10.7% of patients in the probiotic group and 22% in the control group (RR 0.48; 95% CI 0.34–0.7; *P* = 0.0001) ^38^.

Complementing these clinical studies, laboratory studies have also shown that several probiotic strains prevent *Candida* colonization by inhibiting adhesion and biofilm formation, germination, and conversion of yeast to germ (filamentation) ^14, 39^. Overall, the current evidence shows that supplementation of probiotics could be a potentially effective strategy in reducing *Candida* colonization as well as invasive candidiasis in critically ill children.

## Safety of probiotics

Although most commercially available probiotic strains are widely regarded as safe, there are some concerns with respect to safety, particularly in severely debilitated or immunosuppressed patients ^3^. Though *L. rhamnosus* belongs to the normal human rectal, oral, and vaginal mucosal flora, there are a few case reports of liver abscess due to *L. rhamnosus*, lactobacillemia, and infective endocarditis ^40– 46^. Lactobacillus sepsis has been documented in a few reports and was directly linked with the ingestion of probiotic supplements, especially among immunocompromised patients and those with endocarditis ^40^. Kunz *et al.* ^47^ described two premature infants with short gut syndrome who were fed via gastrostomy or jejunostomy and developed *Lactobacillus* bacteremia while taking *Lactobacillus* GG supplements. Land *et al.* ^48^ reported two children with definitive probiotic sepsis: a 4-month-old infant with AAD after cardiac surgery who developed *Lactobacillus* GG endocarditis 3 weeks after commencing *Lactobacillus* GG supplementation and a 6-year-old girl with cerebral palsy and AAD who developed *Lactobacillus* GG bacteremia on day 44 of treatment. The use of *L. rhamnosus* GG in critically ill children was found to have a statistically non-significant trend toward increase in nosocomial infection ^29^. Nonetheless, the risk of infection due to *Lactobacilli* is extremely rare and is estimated to cause 0.05 to 0.4% of cases of infective endocarditis and bacteremia ^49^. There are rare reports of fungemia and septicemia in immunocompromised patients and critically ill patients with the use of *S. boulardii* ^50– 52^. Recently, there have been case reports of *B. longum* bacteremia in preterm infants receiving probiotics ^53, 54^.

Several studies support the general safety of probiotics in a wide range of settings. Manzoni *et al.* ^55^, in a retrospective 6-year cohort study involving VLBW infants, demonstrated that administration of *Lactobacillus* GG as a single dose of 3×10 ^9^ CFU/day from the fourth day of life for 4 to 6 weeks was well tolerated without any adverse effects and that none had bacteremia or sepsis episode attributable to *Lactobacillus* GG. Srinivasan *et al.* ^56^ conducted a prospective study on children admitted to a PICU (n = 28) to establish clinical safety (invasive infection/colonization) of *L. casei Shirota* by bacteriologic surveillance in surface swabs and endotracheal aspirates (colonization) as well as blood, urine, and sterile body fluid cultures. They found no evidence of either colonization or bacteremia with *L. casei Shirota*, and the preparation was well tolerated with no apparent side effects. Simakachorn *et al.* ^57^, in an RCT involving 94 mechanically ventilated children (1 to 3 years), demonstrated that test formula containing a synbiotic blend ( *L. paracasei NCC 2461*, *B. longum* NCC 3001, fructooligosaccharides, inulin, and Acacia gum) was well tolerated.

It has been suggested that the presence of a single major risk factor (immunocompromised state and premature infants) or more than one minor risk factor (cardiac valvular disease, central venous catheter, impaired intestinal epithelial barrier, administration of probiotics by jejunostomy, and probiotics with properties of high mucosal adhesion or known pathogenicity) merits caution in using probiotics because of the risk of probiotics-sepsis ^58^.

Other safety concerns of theoretical importance are genetic transfer of antibiotic resistance from probiotic strains to more pathogenic bacteria in intestinal microbiota (particularly *Enterococcus* and *Staphylococcus aureus*) ^59, 60^, deleterious metabolic activities, and excessive immune stimulation in susceptible individuals ^3, 14^. Many strains of *Lactobacilli* are naturally resistant to vancomycin.

## Future directions

As is evident from many recent studies, probiotics have a promising role in prophylaxis and the treatment of various conditions in critically ill children. However, these results are derived mainly from studies conducted in single centers and are limited by many factors, including small sample sizes, different populations and disease conditions studied, and heterogeneity in the probiotic strains, dose, and duration used. For probiotics to exert their action, it is important that they achieve tight adhesion to intestinal mucosa, and this may be difficult in critical illness. Most of the strains colonize the intestine only after 1 week of consumption, whereas early and effective mucosal adherence is needed to prevent MODS in critically ill children. Well-designed, large multi-center studies are needed for a better understanding of the role of probiotics in critically ill children as well as their pharmacokinetics, mechanisms of action, appropriate dose, administrative regimens, interactions, side effects, risk-benefit potential, and selection of specific probiotics (single-strain or multi-strain), dose, and duration for specific critical care conditions.

## Conclusions

Probiotics have the ability to restore the imbalance of intestinal microbiota and function in critically ill children and have been used for various indications, including the prevention of AAD, HCAIs, VAP, *Candida* colonization, and invasive candidiasis. Safety may be of concern in critically ill, fragile children, as probiotic strains may (albeit rarely) cause bacteremia, fungemia, and sepsis. Well-designed multi-center RCTs are needed to address these issues before the routine use of probiotics is recommended in critically ill children.
